# Intravenous Iron in Patients Hospitalized with Bacterial Infections: Utilization and Outcomes

**DOI:** 10.46804/2641-2225.1176

**Published:** 2024-06-18

**Authors:** Nicolette Centanni, Jennifer Hammond, Joshua Carver, Wendy Craig, Stephanie Nichols

**Affiliations:** a Maine Medical Center Pharmacy, Portland, Maine; b Baystate Medical Center Pharmacy, Springfield, Massachusetts; c Portsmouth Regional Hospital Pharmacy, Portsmouth, New Hampshire; d MaineHealth Institute for Research, Scarborough, Maine; e Westbrook College of Health Professions, University of New England, Westbrook, Maine

**Keywords:** Iron sucrose, iron-deficiency anemia, bacterial infections, patient admission

## Abstract

**Introduction:**

Given the uncertainties related to IV iron therapy and the potential risk of infection, health care providers may hesitate to use this preparation to treat hospitalized patients with bacterial infections, even if clinically indicated. The aim of this study was to examine patterns of prescribing IV iron in patients who were hospitalized and treated for a bacterial infection, and their associated clinical outcomes.

**Methods:**

This retrospective chart review evaluated adult patients who received both IV iron sucrose and antibiotics during the same admission at Maine Medical Center in 2019. Data collected included iron studies, practices for prescribing IV iron, and clinical outcomes. Data were summarized using descriptive statistics.

**Results:**

A total of 197 patients were evaluated. The median duration of antibiotic therapy was 5(4–9) days. Iron and antibiotic administration overlapped in 153(77.7%) patients, with a mean overlap of 2.7(1–7) days. In the 44 patients without overlap, 20(46%) received IV iron before antibiotics. More than half (57%) of infection types involved urinary tract and respiratory systems. Approximately 2% of patients had antibiotic therapy broadened or duration extended, 7% died, and 16% were readmitted within 30 days of discharge.

**Discussion:**

Prior studies evaluating the risk of infection with IV iron published conflicting results. This is the only study that analyzed outcomes in patients receiving IV iron and antibiotics for infection but not undergoing hemodialysis during a hospital admission. Although our findings support that IV iron treatment is safe among patients with concomitant infection and iron deficiency, this finding may not be the case for all clinical subgroups.

**Conclusions:**

This study showed that when patients were administered IV iron in the setting of acute bacterial infection in our facility, most patients did not have negative outcomes.

## 1. Introduction

Iron-deficiency anemia is the number one cause of years lived with disability burden in women and is among the top causes of disability burden worldwide.^1,2^ Iron deficiency may be caused by many factors, including higher iron requirements (eg, pregnancy), low iron intake (eg, unbalanced plant-based diet), chronic blood loss (eg, heavy menses, slow gastrointestinal bleeding), reduced absorption (eg, chronic proton pump inhibitor use, gluten-induced enteropathy, bariatric surgery), or absolute iron deficiency associated with inflammation (eg, chronic kidney disease, heart failure).^1^ Most clinical guidelines define iron-deficiency anemia in populations without chronic kidney disease as having a serum ferritin less than 30 mcg/L.^3^ Iron-deficiency anemia can be complicated in some patients because ferritin is an acute phase reactant. Thus, when ferritin may be higher due to infection or inflammation, greater ferritin thresholds (<100 mcg/L) along with low transferrin saturation (<20%) may have diagnostic value.^1^

Historically, intravenous (IV) iron administration was limited by its propensity to cause hypersensitivity reactions. New preparations, such as iron sucrose, have not shown the same safety concerns. A 2015 meta-analysis including 19253 people from 103 randomized controlled trials (RCT) evaluated the safety of IV iron versus oral iron, intramuscular iron, and IV normal saline (placebo). The meta-analysis did not find a greater risk of severe adverse events, including a severe infusion reaction or mortality, with IV iron versus other forms of iron (oral or intramuscular) or placebo. In contrast, IV iron therapy was associated with a lower risk of gastrointestinal adverse events than oral iron.^4^ Typically, iron repletion can be completed by infusing a total of 1000 mg of iron sucrose divided over 3 to 5 days. This infusion is much faster than oral iron repletion, which can take months.

Untreated iron–deficiency anemia can lead to reduced physical performance and quality of life in adults, as well as cognitive decline and higher mortality in older persons. In people who are pregnant, untreated iron–deficiency anemia may lead to preterm labor, low neonatal weight, and perinatal complications. Iron-deficiency anemia is a negative prognostic factor in chronic heart failure associated with disease progression, lower quality of life, and greater cardiovascular mortality. In chronic kidney disease, anemia is associated with reduced energy and diminished quality of life. Further, iron-deficiency anemia is the most common extra-intestinal manifestation in inflammatory bowel disease. In people with inflammatory bowel disease, anemia symptoms, including weakness, may affect quality of life as much as gastrointestinal symptoms associated with the disease.^2^ As such, IV iron therapy is a guideline-based recommendation for patients with heart failure,^5^ chronic kidney disease,^6^ and colitis.^7^ IV iron therapy also reduced the need for blood transfusions in certain patient populations.^8,9^

Although IV iron therapy is associated with many benefits, iron is a pro-oxidant and important nutrient for bacteria. The administration of IV iron has exacerbated sepsis in lab animals.^10,11^ Certain species of gram-negative rods use unbound iron to facilitate their growth. For example, in vitro studies showed that bacterial growth in *Klebsiella pneumoniae* and *Pseudomonas aeruginosa* cultures increased as transferrin saturation increased, indicating that iron facilitates growth of these bacteria.^12^ The use of IV iron during infection remains controversial as studies have found conflicting results. Two systematic reviews concluded a greater risk of infection with administration of IV iron, whereas other systematic reviews and RCTs reported no risk of infection.^4,8,13–15^

Given the uncertainties related to IV iron therapy and the potential risk of infection, health care providers may hesitate to use this preparation to treat hospitalized patients with bacterial infections, even if clinically indicated. We are unaware of any studies that have explored the patterns of this practice in patients not undergoing hemodialysis. In this exploratory study, we examined the characteristics of hospitalized patients treated for a bacterial infection who were prescribed and administered IV iron and their associated clinical outcomes.

## 2. Methods

### 2.1. Patient cohort

All patients aged 18 years and older who received both iron sucrose (IV iron preparation on institution formulary) and antibiotics during the same admission at Maine Medical Center in 2019 were eligible for inclusion in this retrospective cohort study. Exclusion criteria included patients undergoing hemodialysis; receiving perioperative antibiotics, antibiotics for labor management, antibiotics for chronic prophylaxis, IV iron administered more than 8 days after antibiotic completion, or less than 24 hours of antibiotics (except fosfomycin); and who were not admitted to the hospital or immunocompromised (HIV/AIDS, transplant recipient). We excluded patients undergoing hemodialysis because most studies evaluating IV iron and infection have been conducted in this patient population; yet, in our experience, this concern of infection risk is present for all patients. The study dates were chosen to avoid potential practice changes as a consequence of the COVID-19 pandemic. The study was deemed exempt by the MaineHealth Institutional Review Board.

### 2.2. Data collection

Three contributors (NC - pharmacist, JC - pharmacy student, FW - pharmacy student) collected demographic, clinical, and laboratory data manually from the electronic health record using a standardized protocol. For iron-related laboratory data, we collected the value reported closest to the time before iron sucrose administration. We collected the type of infection categorized as urinary tract, respiratory, endocarditis, skin and soft tissue, bone and joint, meningitis, unspecified sepsis, bacteremia, and abdominal/pelvic. Outcome measures collected included mortality during hospital admission, mortality and/or readmission within 30 days of discharge, receipt of blood transfusion, fever status surrounding IV iron, and duration and scope of antibiotic treatment.

### 2.3. Statistical analysis

Data were summarized using descriptive statistics. Categorical data are shown as No. (%), continuous data are shown as mean ± SD (full range), or median [interquartile range (IQR)] (full range) as appropriate. All analyses were performed using SPSS Statistical Software, version 29 (IBM SPSS Inc., Armonk, NY).

## 3. Results

**Fig. 1.**
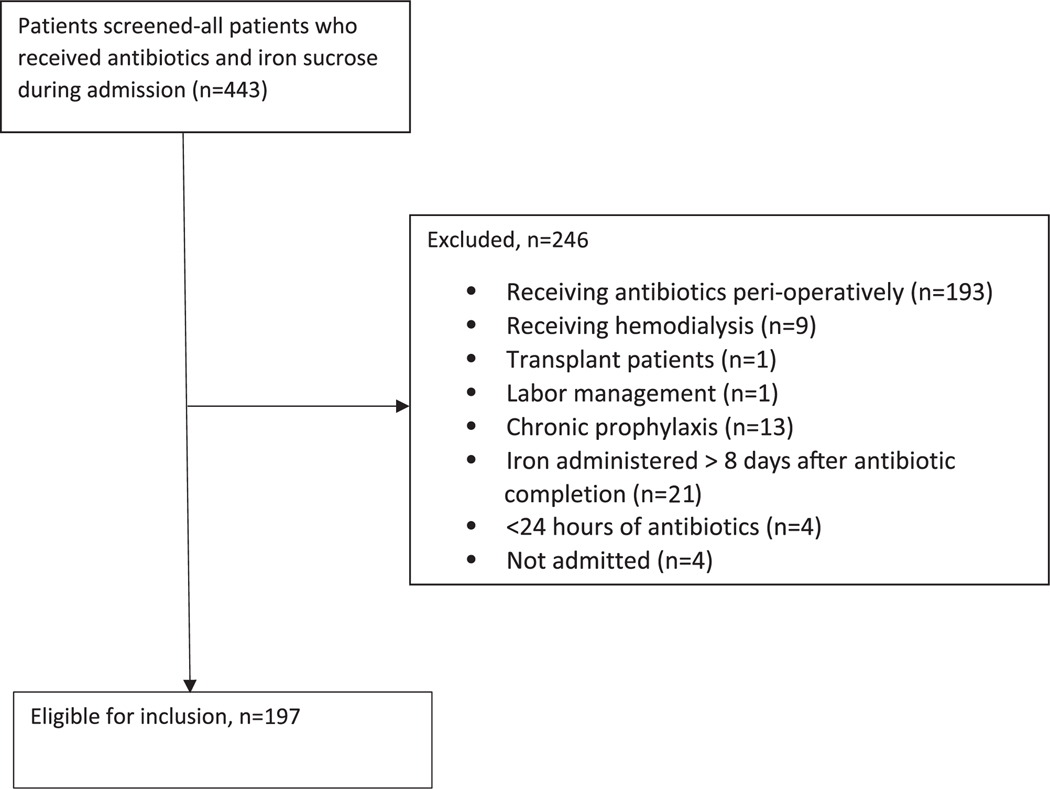
Patient selection flow diagram.

We screened 443 patients who received both IV iron and antibiotics during the same hospital admission. Of these patients, 197 (45%) met inclusion criteria (Fig. 1). Table 1 shows the demographic and clinical characteristics of the study group. The mean age was 67.8±15.7 years. Half of the patients were male (52% male, 48% female), and the median length of stay was 9 days. Most providers (63%) who ordered IV iron specialized in internal or family medicine. Some patients experienced more than 1 infection during their hospital stay, for a total of 202 infections. Of these infections, the most frequent categories were respiratory (68; 33%) or urinary tract (49; 24%) infections.

Table 2 shows laboratory measurements of iron status for the study group before administration of iron sucrose. The median cumulative dose administered during the hospital stay was 600 mg. The mean hemoglobin was 8.6±1.7 g/dL (reference range, male: 13–17.4 g/dL, female: 11.8–15.8 g/dL). Among patients with serum iron measurements available, more than 70% had serum levels less than 30 mcg/dL (reference range, male: 45–160 mcg/dL, female: 30–160 mcg/dL). Serum ferritin levels were available for 83 men (reference range, 30–400 ng/mL) and 83 women (reference range, 30–150 ng/mL). Of these patients 23/83 (28%) men and 19/83 (23%) women had serum ferritin levels above the reference range. Overall, 23/165 (14%) patients had serum ferritin level greater than 500 ng/mL, including 8/165 (5%) with levels greater than 1000 ng/mL. Patients with bacteremia (2/7; 29%) and sepsis (2/5; 40%) were overrepresented among those with ferritin greater than 500 ng/mL.

The median [IQR] duration of antibiotic therapy was 5 [4–9] days (Table 3). Antibiotics and iron sucrose therapy overlapped in 153 (77.7%) patients for a mean duration of 2.7 days. Among 44 patients with no overlap, 24 (55%) received antibiotic therapy first with a median gap of 1 day, and 20 (46%) received iron before antibiotics with a median gap of 2 days.

Table 4 shows clinical outcomes, including mortality and readmission data. A total of 14 (7%) patients died within 30 days of discharge. Among those who died, 10 (71%) appeared to die for reasons unrelated to infection. Among the 32 (16%) readmissions (within 30 days), 22 (69%) were unrelated to infectious causes, and 3 (9%) experienced worsening of their original infection. Blood transfusions were needed for 56 (28%) patients, with 40 (71%) undergoing blood transfusion before IV iron and 12 (21%) after. Of the 2 patients who had a fever within 24 hours before IV iron therapy, neither experienced worsening of that fever. Three (1.6%) patients who were afebrile before IV iron therapy experienced a new fever defined as greater than 38.5°C. Only 5 (2.5%) patients had an extended duration, and 4 (2%) had broadened antibiotic therapy after IV iron therapy.

## 4. Discussion

The risk of administering IV iron during an acute infection remains unknown and must be weighed against the risks of untreated iron-deficiency anemia. Expert opinions, such as the published KDOQI (National Kidney Foundation-Kidney Disease Outcomes Quality Initiative) in the KDIGO (Kidney Disease: Improving Global Outcomes) 2021 guideline for anemia in chronic kidney disease, concluded that data are equivocal for determining if the risk of infection or worsened outcomes are greater with IV iron. These guidelines state that individual factors, such as severity of infection, need to be considered.^16^

During an infection, iron sequestration is enhanced by lactoferrin as well as peptides, such as hepcidin, and various cytokines. Like humans, microorganisms require iron for cellular processes. In iron-depleted human hosts, pathogens use pathways for heme uptake and non-heme iron acquisition. One of those pathways is the release of siderophores that scavenge free ferric iron and then undergo reuptake into cells. The mechanism of this interaction between iron and bacteria has even been a target of new antibiotic therapy. Cefiderocol, approved in 2019, is the first in its class as an injectable siderophore cephalosporin used for treating complicated urinary tract infection and hospital acquired pneumonia. This therapy is a combination of a catechol-type siderophore and a cephalosporin that chelates extracellular iron and facilitates uptake into bacterial cells.^17,18^

A 2013 systematic review and meta-analysis of 75 trials included 10605 patients. This analysis found that although IV iron reduced the need for blood transfusions, IV iron was associated with a significantly greater risk of infection [relative risk (RR), 1.33; 95% CI, 1.1–1.65] than oral or no iron supplementation.^8^ In 2021, another systematic review and meta-analysis of 154 RCTs including 32762 patients found that IV iron was associated with a greater risk of infection than oral iron or no iron supplementation (RR, 1.16; 95% CI, 1.03–1.29). This meta-analysis included a heterogeneous population but found no effect on mortality or hospital length of stay.^14^

On the contrary, many studies found no higher risk of infection with IV iron administration. The 2015 meta-analysis discussed previously also evaluated the risk of infection with IV iron therapy. This analysis found no greater risk of serious infections with IV iron in this patient population (RR, 0.96; 95% CI, 0.63–1.46).^4^ In the largest randomized, multi-center trial (FIND-CKD) including patients with chronic kidney disease that do not depend on dialysis, 626 patients were randomly assigned to receive either oral or IV iron with differing ferritin targets. No difference in serious adverse events [75/304 (24.7%) with IV vs 59/312 (18.9%) with oral iron] or severe infections [11/304 (3.6%) with IV vs 12/312 (3.8%) with oral iron] were observed between the groups.^14^ Also, a nationwide cohort-based study in 1410 patients undergoing hemodialysis found that IV iron supplementation did not increase short-term infection risk (OR, 1; 95% CI, 0.75–1.33).^19^ An RCT of critically ill patients found no difference in the infection rate between groups who received iron sucrose 3 times weekly vs placebo for 2 weeks.^15^ Furthermore, some studies suggested that the risk of infection is not significantly greater when serum ferritin is less than 500 ng/mL.^10^

The only other study that looked specifically at patients hospitalized for infection and receiving IV iron is a retrospective observational cohort study from 2015.^20^ The included patients all depended on hemodialysis, and 2463 patients received IV iron at any point during their hospitalization. The authors concluded that receipt of IV iron was not associated with higher 30-day mortality (OR, 0.86; 95% CI, 0.74–1.00) or readmission for infection or death within 30 days of discharge (OR, 1.08; 95% CI, 0.96–1.22).^20^ We are not aware of any other study that includes patients who were hospitalized but not undergoing dialysis while receiving therapy for bacterial infections.

We found that internal and family medicine had the highest incidence of prescribing iron sucrose to patients with an acute infection. This finding could be due to a higher comfort level with the paucity of data related to the risks, or that these providers may care for a higher number of patients with active infection than other disciplines. We also found that most of the time, administration of iron sucrose and antibiotics overlapped for around 3 days. When they did not overlap, antibiotics were usually given first, followed by iron sucrose, indicating that treatment of infection was prioritized over iron deficiency. Most patients had a ferritin below 500 ng/mL, which is a proposed threshold for a greater risk of infection with IV iron administration. Other authors have proposed a threshold of more than 1000 ng/ml.^14^

We found that most patients had an overlap of antibiotics and IV iron during treatment. This finding means the infection was still actively being treated while IV iron was administered. Of the patients that did not have an overlap of IV iron and antibiotics, 55% had completed antibiotics before IV iron, which suggests hesitancy in administering IV iron with an active infection.

Because this retrospective study did not have a comparator group, we cannot infer that any infection-associated outcomes were caused by IV iron treatment. Rather, our study provides descriptive information about outcomes that might occur among patients admitted for infection and treated for incidental iron deficiency with IV iron, with 28% also requiring a blood transfusion. We found a low incidence of infection-related adverse outcomes. For example, only 3 (1.6%) patients became febrile after IV iron administration. Fourteen (7%) patients died within the study period, of which 4 died from respiratory failure, which could have been related to infection. The other 10 patients died from causes seemingly unrelated to infection. Similarly, among the 32 patients readmitted to the hospital within 30 days, most were readmitted for reasons unrelated to infection, and 31% (5% of the overall study group) were readmitted due to infectious causes.

Although our findings support that IV iron treatment is safe among patients with concomitant infection and iron deficiency, this safety may not be the case for all clinical subgroups. Future studies could explore outcomes in patients with immunodeficiency, severe infections, and/or those with markedly elevated amounts of serum ferritin.

The limitations of this study include its retrospective design and lack of a comparator group. Also, given the study design, data were only available for fields documented in the electronic health record. Therefore, information about the clinical decision-making process during the patient’s stay was limited.

## 5. Conclusions

Our study showed that IV iron is prescribed and administered to treat anemia in the setting of acute bacterial infections at our institution in a variety of patients, including many older adults. A large majority of patients did not appear to have negative outcomes after receiving IV iron. Future studies are needed to determine the risk of worsening infection in patients who are hospitalized and given IV iron, both generally and in vulnerable clinical subgroups. In addition, future studies might investigate whether factors such as ferritin can help predict the safety of IV iron in the context of an acute infection.

## Figures and Tables

**Table 1.** Demographic and clinical characteristics of the study group (N = 197).

Variable	Value
Age, mean±SD (full range), y	67.8±15.7 (20–95)
Sex, No. (%)	
Female	95 (48.0)
Male	102 (52.0)
Provider group, No. (%)	
Internal/Family medicine	128 (63.4)
Cardiology	40 (20.3)
Surgery	18(9.1)
Intensive care unit	2 (1.0)
Nephrology	2 (1.0)
Other	7 (3.5)∗
Infection type, No. (%)^†^	
Respiratory	68 (32.9)
Urinary tract infection	49 (23.7)
Skin/Soft tissue	29 (14.4)
Abdominal/Pelvic	27 (13.4)
Bone/Joint	10 (4.8)
Bacteremia	7 (3.4)
Endocarditis	6 (3.0)
Sepsis (unspecified)	5 (2.5)
Meningitis	1 (0.5)

* Includes n=3 obstetrics and n=1 each of oncology, pediatrics, psychiatry, and pulmonology.

† n=192 had 1 infection listed and n=5 had 2 infections listed.

**Table 2.** Iron studies (N = 197).*

Variable	Value
Cumulative iron dose	
No. (%)	197 (100)
Median [IQR] (full range), mg	600 [400–1000] (100–1100)
Hemoglobin before IV iron^†^	
No. (%)	197 (100)
Mean±SD (full range), g/dL	8.6±1.7 (2.6–14.9)
Serum iron	
No. (%)	171 (86.8)
Median [IQR] (full range), *μ*g/dL	23 [16–30] (9–286)
Serum iron category, *μ*g/dL, No (%)	
No. (%)	171 (86.8)
<10	5 (2.9)
10–19	69 (40.4)
20–29	52 (30.4)
30–39	20 (11.7)
40–49	13 (7.6)
50–59	4 (2.3)
≥60	8 (4.7)
Iron binding capacity	
No. (%)	166 (84.3)
Mean±SD (full range), *μ*g/dL	266±89 (107–543)
Transferrin saturation, %	
No. (%)	166 (84.3)
Median [IQR] (full range)	9[6–14] (3–71)
Serum ferritin	
No. (%)	165 (83.8)
Median [IQR] (full range), ng/mL	96 [38–316] (3.3–2540)
Serum creatinine^‡^	
No. (%)	197 (100)
Median [IQR] (full range), mg/dL	1.0 [0.7–1.5] (0.4–5.7)

Abbreviations: IQR, interquartile range; IV, intravenous.

* Results received before the intervention.

† Value closest to time of IV iron administration.

‡ Within 1 week of IV iron.

**Table 3.** Treatment characteristics of the study group (N = 197).

Variable	Value
Total duration of inpatient antibiotics, median [IQR] (full range), days	5 [4–9] (1–148)
Overlap between IV iron and antibiotic treatmen No. (%)	153 (77.7)
Mean±SD (full range), days	2.7 ± 1.4 (1–7)
No overlap between IV iron and antibiotic treatment, No. (%)	44 (22.3)
Antibiotics before IV iron	
No. (%)	24/44 (54.5)
Time between antibiotics and IV iron, median [IQR] (full range), days	1 [1–2] (1–8)
IV iron before antibiotics	
No. (%)	20/44 (45.5)
Time between IV iron and antibiotics, days, median [IQR] (full range)	2 [1–3.8] (1–27)

Abbreviation: IQR, interquartile range.

**Table 4.** Clinical outcomes (N = 197).

Variable	Value, No. (%)
30-day mortality	14 (7.1)*
30-day re-hospitalization within MaineHealth	32 (16.2)
Worsened original infection	3 (9.4)
New infection	7 (21.9)
Not related to infection	22 (68.8)
Blood transfusion during hospital stay	56 (28.4)
Before IV iron	40 (71.4)
After IV iron	12 (21.4)
During IV iron	4 (7.1)
Fever (>38.5°C) within 24 h before IV iron	2 (1.0)
Worsened in 24 h after IV iron^†^	0/2 (0.0)
New fever (>38.5°C) within 24 h after IV iron	3/193 (1.6)^‡^
Antibiotic treatment longer than guideline recommended or initial plan	5 (2.5)
Antibiotic coverage broadened after IV iron started	4 (2.0)

Abbreviations: IV, intravenous.

* Includes 4 respiratory failure; 3 heart failure; 2 unknown; and 1 each of cardiac arrest, dehydration/hypernatremia, hepatic malignancy, spinal cord injury after trauma (fall), and renal failure subsequent to cardiac arrest.

† Increase in temperature of at least 1°C.

‡ Fever defined as >38.5°C.
